# Does the Side Matter? Medial vs Lateral Ankle Dorsiflexion Measurements During the Silfverskiöld Test in Children

**DOI:** 10.1177/10711007251351317

**Published:** 2025-07-20

**Authors:** Suki Liyanarachi, Cecilie Jansen Basma, Olav Andreas Foss, Christian Reidar Øye, Ketil Jarl Holen, Håkon Langvatn

**Affiliations:** 1 Norwegian University of Science and Technology (NTNU), Trondheim, Norway; 2 St Olavs Hospital, Trondheim University Hospital, Trondheim, Norway

**Keywords:** gastrocnemius, Silfverskiöld, ankle dorsiflexion, goniometry, pediatric

## Abstract

**Background:**

Isolated gastrocnemius tightness is associated with several foot conditions. The Silfverskiöld test examines for such tightness, and when assessing passive ankle dorsiflexion it is important to perform the examination with the proper technique. Several studies have reported reproducible examination techniques, yet none, to our knowledge, have evaluated whether measurement side—medial or lateral—affects dorsiflexion values. Understanding measurement consistency is crucial for clinical practice and research standardization. We have undertaken a study investigating whether this is of importance and assessed the repeatability of ankle dorsiflexion measurements.

**Methods:**

We performed an exploratory cross-sectional examination of 145 pediatric patients (290 feet) with a 2-person 2-hand Silfverskiöld test. Masked, repeated goniometric measurements were undertaken along both the medial and lateral axis of the leg and foot.

**Results:**

There was a small systematic difference between medial and lateral measurements where lateral measurements were on average almost 2 degrees more in equinus. The repeatability coefficient for all repeated measurements ranged from 5.1 to 5.5 degrees. The intraclass correlation coefficient between medial and lateral measurements was excellent (ICC = 0.97).

**Conclusion:**

We found a small systematic difference of 2 degrees between medial and lateral measurements that was less than the repeatability coefficient. The repeatability coefficient was 5 degrees, meaning that for ankle dorsiflexion measurements it is unlikely that a second measurement will differ more than 5 degrees. We do not believe that the statistically significant small difference between medial and lateral measurements is clinically relevant; however, it makes sense to consistently measure ankle dorsiflexion on either the medial or lateral side.

## Graphical abstract

**Graphical Abstract:**
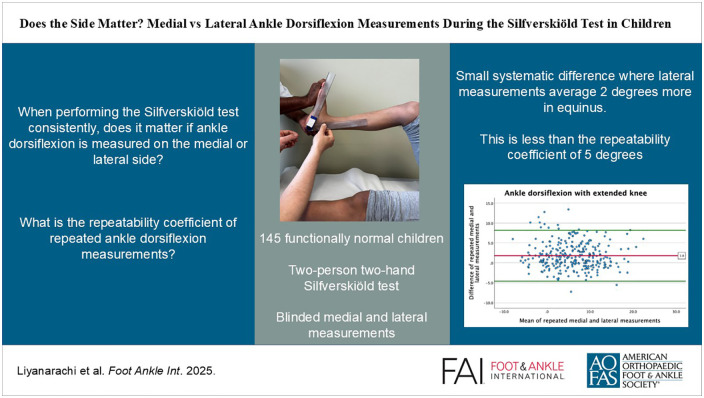
This is a visual representation of the abstract.

**Level of Evidence:** Level II, diagnostic.

## Introduction

It is important to have a simple and reliable technique for measurement of passive ankle dorsiflexion. Passive ankle dorsiflexion changes with knee positioning. The Silfverskiöld test examines this discrepancy. Reduction in passive ankle dorsiflexion with the knee extended, relative to when the knee is flexed, is due to the gastrocnemius muscle.^29^ Isolated tightness of the gastrocnemius muscles has been associated with several foot conditions in adults.^10,18,20,26^ Apart from cerebral palsy and flat foot, there is limited literature investigating this association in children; however, some authors suggest an association with heel apophysitis, toe walking, and other foot complaints.^2,30^

The Silfverskiöld test may seem like a simple clinical test; however, performing it consistently may be challenging as several factors need to be addressed. Patient positioning, knee position, the amount of dorsal flexion force applied, and landmarks used for measurements must be standardized.^4,11,14,16^ The examiner must also ensure that only passive motion is assessed, where the patient should not aid or resist actively. Additionally, it is important to correct for compensatory mid- and hindfoot motion as demonstrated in several previous publications, using a 2-hand technique.^5,8,21,24^ Traditionally a 2-arm goniometer has been used to measure ankle dorsiflexion. Previous studies have demonstrated variations in the reliability of goniometric measures, which reflects the variety of examination techniques used.^6,28,31,32^ Some authors have tried to address variations in technique by building jigs that control and standardize foot and knee positioning and the force applied.^9,16,17^ Several publications have shown that such devices provide more consistent measurements than the clinical test. To our knowledge, such devices are not validated in the pediatric population. For children of different age, size, and maturity, the clinical Silfverskiöld test is still the examination method of choice, where all the factors listed above should be addressed.^14^

When assessing ankle dorsiflexion in children, is it of importance which landmarks are used for measurements? Medial and lateral landmarks differ as the leg axis along the fibula may be different from the medial leg axis. Additionally, the medial side of the foot may elevate compared with the lateral side when the mid- and hindfoot is locked in varus using the 2-hand dorsiflexion technique. It is therefore conceivable that there may be a demonstrable, systematic discrepancy using the medial foot axis compared with the lateral foot axis when assessing ankle dorsiflexion on the same subject by the same examiners. Most publications use lateral landmarks for goniometric positioning or omits specifying how measurements were performed,^1,12,19^ whereas only a few specify using medial landmarks.^22,27^ Presumably, goniometers are most often placed laterally because lateral landmarks are easily identifiable.

Assuming that the Silfverskiöld test is performed correctly, we wanted to undertake an exploratory study assessing if the side used for the goniometric measurements of ankle dorsiflexion was important. A second aim was to evaluate the repeatability of these measurements in children.

## Methods

The study was approved by the regional ethics committee (526706 REK midt), and all children were included after informed parental consent.

We performed an exploratory cross-sectional study on children attending the pediatric orthopaedic outpatient clinic at a large teaching hospital, over a 16-month period. All recruited patients were functionally healthy children with normal clinical variability between 4 and 14 years of age. This age range was chosen to ensure that the children would be old enough to comply with instructions, and still not fully grown. Most included children (111) were new referrals with leg and foot complaints such as heel apophysitis, flat feet, accessory navicular bone and nightly leg pains. Additionally, a smaller group of children (34) attending fracture clinic for upper extremity fracture follow-up without foot and leg complaints were included. All included patients were found to be healthy on clinical examination, with normal functioning lower extremities. Children with fixed foot deformities or lower extremity malalignment, tarsal coalitions, neuro-orthopaedic conditions, lower extremity fracture, infection, or tumor sequelae were excluded.

The children were examined in the outpatient clinic under normal clinical circumstances by 3 experienced pediatric orthopaedic clinicians. We used the same standardized Silfverskiöld test as in our previous publication on schoolchildren to assess passive ankle dorsiflexion.^14^ Time and care was taken to make the child relax in a supine, semireclined position. The test was performed as a 2-hand technique controlling for mid- and rearfoot motion, avoiding midfoot abduction and locking the hindfoot with varus. Care was taken to avoid that the child aided or resisted dorsiflexion actively, which was best seen and felt by the examiner. Visual contraction of dorsal extensors, palpable contraction of calf muscles or contraction of the patella were useful aids for the examiner to ensure that true passive motion was being assessed, when applying dorsiflexion force. The ankle was dorsiflexed with flexed and extended knee with enough force to achieve an end point of full range of motion, maintaining a comfortable, relaxed child. Applying increased force making the child uncomfortable was avoided as this would cause compensatory contractures of leg muscles, which the examiner had to be aware of.

Of the 3 examining clinicians, one performed the clinical dorsiflexion examination on all children and registered digital dorsiflexion recordings, while one of the others laid the goniometer. The degree of ankle dorsal flexion was measured with a simple 2-arm goniometer with a digital display (Medtronic), with surface markings removed and the display covered by tape to ensure masked measurements. After each dorsiflexion measurement, the tape was removed and the angle recorded. Medial and lateral dorsiflexion measurements were undertaken with the knee flexed and fully extended, twice consecutively. Clinician 1 performing the Silfverskiöld test was positioned at the end or the right side of the patient, while the other clinician was on the left side. The order of examination was fixed beginning with medial measurements on the right leg and lateral measurements on the left leg, which was then repeated. Clinicians then changed side and continued with medial measurements on the left leg and lateral measurements on the right leg, which was also repeated. Apart from repeated measurements, the examinations were performed in the same manner as under normal clinical circumstances. The mid-axis of the leg was medially defined as approximately 1 cm posterior of the medial border of the tibia, and laterally along the fibula. For the foot, the visual axis of weightbearing was used for both sides, where the goniometer was laid from the load area of the plantar surface of the heel to either the load area under the first or fifth metatarsal head (see supplementary material).

The statistical analyses were framed as exploratory and were performed using IBM SPSS Statistics (version 29; IBM Corp, Armonk, NY). Visual inspection of histograms showed the ankle measurements to be normally distributed. Consequently, data were presented as mean and SD.

Bland-Altman plots are presented with the mean differences between the corresponding dorsiflexion measurements, obtained laterally or medially, and the 95% limits of agreement.^3^ The repeatability between the corresponding measurements were also calculated according to the method of Bland and Altman where the repeatability coefficients are twice the SD of the differences between the corresponding sets of measurements.

Generalized linear mixed models were used to account for data dependency caused by repeated measures within each child. To describe the possible differences in ankle dorsiflexion when measurements were obtained along the medial or lateral axis of the foot, statistical models with 3 levels were used. Measurements (laterally or medially along the axis of foot) were nested within the side (left or right) and further nested within children. Further, the intraclass correlation coefficient (ICC) was calculated using a 2-way random effects model.

## Results

One hundred forty-five children were recruited. Both feet were examined in all children, and the standardized Silfverskiöld test was performed on 290 feet, with 8 registered measurement values per foot.

The mean age of the included patients were 10 years (range 4-14 years, and 54% were boys. Of the clinicians performing the goniometry, clinician 2 performed 59% of the measurements.

### Difference Between Medial and Lateral Measurements

Table 1 presents the comparison between medial and lateral measurements. Lateral measurements were on average more pronounced in equinus with both flexed and extended knee (Table 1).

**Table 1.** Comparison of Medial and Lateral Measurements.^a^

		Mean Ankle Dorsiflexion	Difference of Mean (95% CI)	*P* Value
Medial	Knee flexion 1 Knee flexion 2	28.5	1.9 (1.6, 2.2)	<.001
Lateral	Knee flexion 1 Knee flexion 2	26.6		
Medial	Knee extension 1 Knee extension 2	6.9	1.8 (1.5, 2.1)	<.001
Lateral	Knee extension 1 Knee extension 2	5.1		

a This table presents the mean of combined measurements performed medially or laterally with flexed and extended knee, the difference of the combined mean with 95% CI and the statistical results of our model in 3 levels testing the difference between medial and lateral measurements.

**Figure 1.**
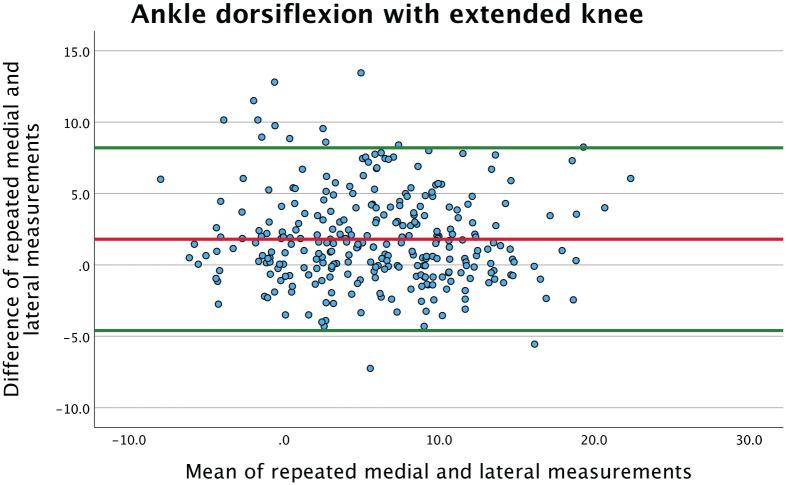
Bland-Altman plot presenting the mean of the 2 lateral and the 2 medial ankle dorsiflexion measurements with extended knee. The red line represents the mean of the difference, showing a systematic bias where on average lateral measurements are 1.8 degrees more in equinus than medial. [See online article for color figure.]

The agreement between medial and lateral measurement was visualized in a Bland-Altman plot. With extended knee, the mean of the difference was on average 1.8 degrees more in equinus measured on the lateral side compared with the medial side (Figure 1). The Bland-Altman plot was similar for measurements with flexed knee.

**Figure 2.**
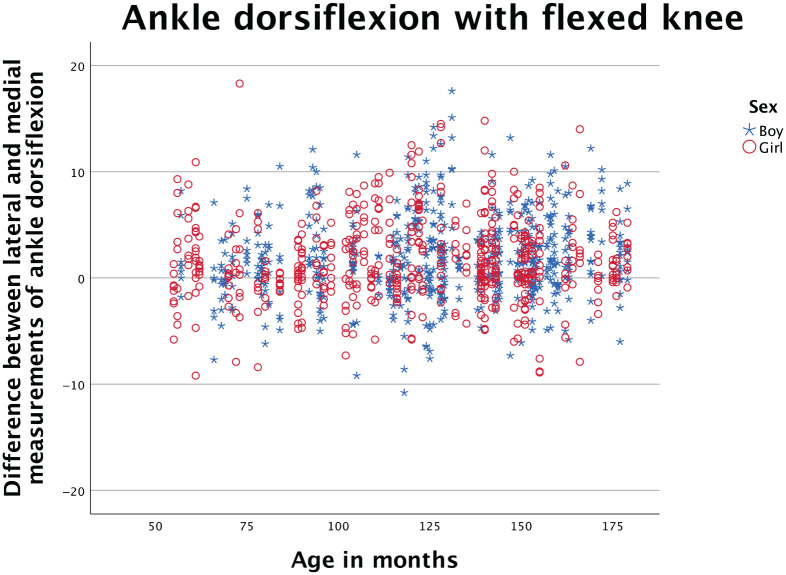
Scatterplot presenting difference between lateral and medial measurements of dorsiflexion with flexed knee, plotted against age. Sex-specific markers are used. Based on visual inspection, the scatterpoints are evenly distributed and there is no change in the difference according to age or sex.

Figure 2 presents the difference of lateral and medial measurements compared to age, using sex-specific markers. Based on visual inspection of the scatterplot there is no change in the difference throughout the age range that we have examined, and no systematic difference between sex.

### Repeatability

The mean of repeated medial and lateral measurements with flexed knee and extended knee are presented in Table 2. For all 4 sets of repeated measurements, the mean of the differences were close to zero, indicating that there is little systematic difference with repeated measurements. The SD of the difference was comparable in all 4 sets at 2.6-2.7.

**Table 2.** Repeated Medial or Lateral Measurements.^a^

		Mean Ankle Dorsiflexion	Difference of Mean	SD Difference	Repeatability Coefficient
Medial	Knee flexion 1	28.6	0.2	2.6	5.1
	Knee flexion 2	28.4			
Medial	Knee extension 1	7.0	0.2	2.6	5.3
	Knee extension 2	6.8			
Lateral	Knee flexion 1	26.7	0.3	2.7	5.5
	Knee flexion 2	26.4			
Lateral	Knee extension 1	5.1	0.0	2.7	5.4
	Knee extension 2	5.1			

a This table presents the mean ankle dorsiflexion measured 8 times, the difference between the mean of repeated measurements, the SD of the differences, and the repeatability coefficient for each set of repeated measurements.

**Figure 3.**
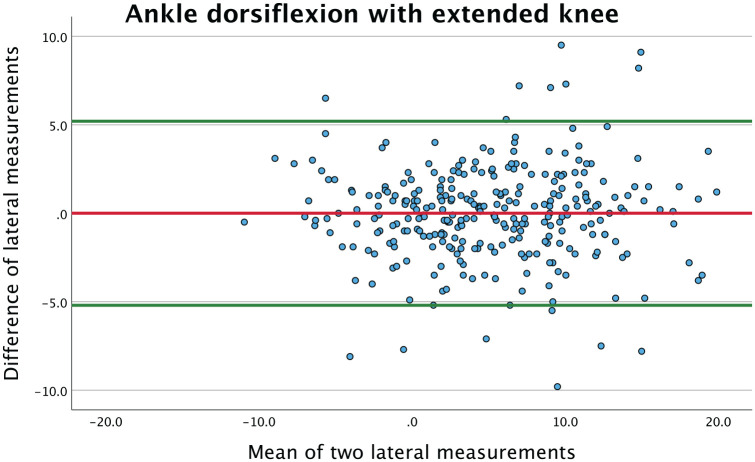
Bland-Altman plot presenting the mean of repeated lateral measurements of ankle dorsiflexion with extended knee plotted against the difference. The red line represents the mean of the difference, showing a value close to zero. The green lines represent the limits of agreement (95% CI). [See online article for color figure.]

This is further illustrated in a Bland-Altman scatterplot (Figure 3) where the differences between the repeated lateral measurements with extended knee are plotted against their averages. This plot demonstrates that the mean of the differences were close to zero and that there were no relations between differences and the magnitude, based on visual evaluation. The scatter distribution is evenly distributed between +5 and −5 degrees, which was similar for the remaining 3 sets of repeated measurements.

The repeatability coefficient of ankle dorsiflexion with flexed knee was 5.1 degrees measured medially and 5.5 degrees measured laterally. With extended knee, the repeatability coefficient was 5.3 degrees measured medially and 5.4 degrees measured laterally.

The ICC for the correlation between corresponding medial and lateral measurements was ICC 0.97.

## Discussion

In our exploratory study in children comparing lateral and medial measurements, our results show a small systematic difference with lateral measurements averaging nearly 2 degrees more in equinus compared to medial measurements. This applies to ankle dorsiflexion with both flexed and extended knee. Although this difference was statistically significant, it falls well within our measurement repeatability coefficient of 5 degrees, suggesting limited clinical relevance for individual patient assessments.

In contrast, no systematic difference is found when comparing repeated medial and lateral measurements respectively. When testing our repeatability of measuring ankle dorsiflexion on either the medial or lateral side, our repeatability coefficient is close to 5 degrees. This means that at the individual level we can expect that a second measurement of dorsiflexion on the same patient on the same side will not differ more than 5 degrees from the first measurement in 95% of cases. In our opinion, this coincides with our clinical experience, as differences below 5 degrees are difficult to separate and therefore of less clinical relevance. An ICC value of 0.97 as presented herein represents excellent agreement based on conventional thresholds^15^ and was to be expected because it describes the overall correlation between the medial and lateral measurements.

The 2 degrees of increased equinus in lateral measurements was statistically significant at the group level (*P* < .001); however, this difference is less than the repeatability coefficient of 5 degrees. We therefore believe this finding is clinically negligible and not of clinical significance at the individual level. Our results also demonstrate that the consistency of medial or lateral measurements are comparable, as the repeatability coefficient is similar. In the clinical setting, choosing to consistently measure on the medial or lateral side should thus not be of any importance. Our preference is performing the goniometry on the medial side, as the examiner performing the Silfverskiöld test can then stand on the ipsilateral side of the leg being examined. Despite the lack of clinical significance, our findings could have importance when comparing measurements at the group level, and as there is a small difference it makes sense to be consistent in how one performs ankle dorsiflexion measurements.

We do not know the cause for the small discrepancy between medial and lateral measurements. One possible explanation may be the difference in foot axis when measuring ankle dorsiflexion as one applies an adductional force avoiding midfoot abduction and locking the hindfoot in varus. This 2-hand examination technique may create a slight supination elevating the medial column of the foot more than the lateral column. There may also be differences of the medial and lateral long axis of the leg when using the tibia or fibula as reference.

In our population we have measured ankle dorsiflexion adhering to a set protocol by experienced clinicians. We were aware of the need to neutralize the hindfoot and avoid midfoot abduction. The difference between medial and lateral measurements could be more pronounced when such a protocol is not adhered to. The repeatability coefficient may also be different in less experienced examiners not accounting for the pitfalls of the clinical examination.

We are aware of criticism that ankle dorsiflexion measurements may be difficult to reproduce consistently.^9,17^ Previous studies have demonstrated varying reproducibility of measurements from poor to good correlation. This highlights the diversity in examination technique and lack of standardized examination protocol.^28,31^ If a set protocol is adhered to, a good intrarater agreement can be achieved.^23^ Several authors have designed and used specific devices to consistently and accurately measure ankle dorsiflexion.^9,12,17^ Results show that such devices provide an opportunity to consistently measure gastrocnemius tension in the adult population. As far as we know, these devices are not validated for children. We do not believe that it will be possible to use a similar protocol and device when examining children of varying age, shape, size, and maturity. For instance, it is important that the child is relaxed to measure passive dorsiflexion. An uncomfortable, scared, or rushed child will easily aid or resist the dorsiflexion motion, giving false estimates. The same is important when applying the dorsiflexion force. The examiner must apply enough force on the passive extremity to achieve full range of motion without causing discomfort. If the child is uncomfortable, reciprocal agonist or antagonist muscle activity will affect the results. In our experience, full dorsiflexion with flexed knee is rarely challenging, but limitations to dorsiflexion by the gastrocnemius muscle with fully extended knee could provide discomfort if the examiners are not attentive. This would be hard to ensure when using specific devices for measurement. The purpose of our exploratory study was to examine our clinical examination on real patients, performed in a similar way as we would have done clinically. We purposefully chose to only examine healthy children with normal-functioning lower extremities, some with increased ankle range of motion and other with limitations, where the examination could be more challenging. We have demonstrated that ankle dorsiflexion measurements in children can be repeated with a repeatability coefficient of 5 degrees, when the examination is performed following a standardized technique.

We are also aware of authors advocating weightbearing lunge test of ankle motion.^13,25^ This does not examine the limitations of dorsiflexion due to the gastrocnemius muscle in the same manner. Weightbearing ankle dorsiflexion may differ from passive dorsiflexion.^7^ Additionally, we do not believe that a weightbearing examination will be possible to undertake in a standardized manner particularly in the youngest children, while controlling for knee positioning and foot abduction.

### Strengths and Weaknesses

Our intention was not to measure the Silfverskiöld test as accurately as possible in a lab or research setting, but rather assess the repeatability of our test in a clinical setting. Our study subjects represent a range of pediatric patients with different age and size, as one would expect in a clinical setting. We believe this is a major strength of our study. A limitation of our study is that we have not included children with pathologic findings of lower extremity function such as children with CP, and do not know what our results would have been for these patients. Another possible challenge is that the Silfverskiöld test has many nuances making the examination vulnerable to variations that may affect measurement results. This includes patient positioning, measuring only passive motion and ensuring that the dorsiflexion force applied is comparable. Yet we believe that performing this clinical test in a standardized manner as presented is the only reliable way to perform this examination in children. Furthermore, we have only examined the repeatability of the examination by the same examiners in the same setting. Testing the interrater reproducibility of measurements by different examiners or in different settings was not part of our study objectives and is thus an important limitation. We also did not assess intrarater reliability over time, limiting conclusions about test-retest reproducibility. Additional limitations include the lack of test-retest reliability assessment over different time points, potential selection bias from recruiting primarily from orthopaedic clinics, and the exclusion of children with pathologic conditions, which limits generalizability to the broader pediatric population. Future studies should examine the interrater reproducibility because clinical measurements of ankle range of motion often involve multiple providers. Even though our goniometric measurements were collected masked, the examination itself was not masked, which would be difficult to do in children. There is also a potential for order effect bias because there was a fixed order of measurements, introducing a minor measurement bias. Future work should randomize the testing sequence to avoid this bias. Finally, our technique required 2 examiners to measure the range of motion as objectively as possible, where one performed the dorsiflexion maneuver and the other laid the goniometer arms. This may differ from clinical workflows, limiting generalizability. The actual dorsiflexion examination is, however, performed as one typically would do in the clinical setting.

## Conclusion

We did not find any clinically significant difference between medial and lateral measurements of ankle dorsiflexion measured in the same patient, by the same examiner. Although clinically negligible, we found a small but statistically significant difference of 2 degrees indicating that clinicians and especially researchers should be consistent with their measurements and always measure on the same side. Our findings support the reliability of the Silfverskiöld test when performed using standardized technique, with a repeatability coefficient of approximately 5 degrees for both medial and lateral approaches. Furthermore, researchers should consider reporting which side they measure from and state that measurements were performed consistently.

## Supplemental Material

sj-docx-4-fai-10.1177_10711007251351317 – Supplemental material for Does the Side Matter? Medial vs Lateral Ankle Dorsiflexion Measurements During the Silfverskiöld Test in Children Supplemental material, sj-docx-4-fai-10.1177_10711007251351317 for Does the Side Matter? Medial vs Lateral Ankle Dorsiflexion Measurements During the Silfverskiöld Test in Children by Suki Liyanarachi, Cecilie Jansen Basma, Olav Andreas Foss, Christian Reidar Øye, Ketil Jarl Holen and Håkon Langvatn in Foot & Ankle International

sj-jpg-2-fai-10.1177_10711007251351317 – Supplemental material for Does the Side Matter? Medial vs Lateral Ankle Dorsiflexion Measurements During the Silfverskiöld Test in Children Supplemental material, sj-jpg-2-fai-10.1177_10711007251351317 for Does the Side Matter? Medial vs Lateral Ankle Dorsiflexion Measurements During the Silfverskiöld Test in Children by Suki Liyanarachi, Cecilie Jansen Basma, Olav Andreas Foss, Christian Reidar Øye, Ketil Jarl Holen and Håkon Langvatn in Foot & Ankle International

sj-jpg-3-fai-10.1177_10711007251351317 – Supplemental material for Does the Side Matter? Medial vs Lateral Ankle Dorsiflexion Measurements During the Silfverskiöld Test in Children Supplemental material, sj-jpg-3-fai-10.1177_10711007251351317 for Does the Side Matter? Medial vs Lateral Ankle Dorsiflexion Measurements During the Silfverskiöld Test in Children by Suki Liyanarachi, Cecilie Jansen Basma, Olav Andreas Foss, Christian Reidar Øye, Ketil Jarl Holen and Håkon Langvatn in Foot & Ankle International

sj-pdf-1-fai-10.1177_10711007251351317 – Supplemental material for Does the Side Matter? Medial vs Lateral Ankle Dorsiflexion Measurements During the Silfverskiöld Test in Children Supplemental material, sj-pdf-1-fai-10.1177_10711007251351317 for Does the Side Matter? Medial vs Lateral Ankle Dorsiflexion Measurements During the Silfverskiöld Test in Children by Suki Liyanarachi, Cecilie Jansen Basma, Olav Andreas Foss, Christian Reidar Øye, Ketil Jarl Holen and Håkon Langvatn in Foot & Ankle International
