# Supplementary material for: Does the Side Matter? Medial vs Lateral Ankle Dorsiflexion Measurements During the Silfverskiöld Test in Children
Source: Foot Ankle Int. 2025 Jul 20;46(10):1176–83. doi: 10.1177/10711007251351317 (PMC12534877; doi:10.1177/10711007251351317)
Supplement: sj-docx-4-fai-10.1177_10711007251351317 – Supplemental material for Does the Side Matter? Medial vs Lateral Ankle Dorsiflexion Measurements During the Silfverskiöld Test in Children [file sj-docx-4-fai-10.1177_10711007251351317.docx]

Supplemental figure 1. Photograph illustrating goniometric placement on the medial side while performing the two-hand examination technique of ankle dorsiflexion with flexed knee. The examiner performing the ankle dorsiflexion locks the hindfoot in varus, avoids midfoot abduction and applies dorsiflexion force to the whole foot, whilst still exposing the medial aspect of the foot for consistent goniometer placement. The goniometer arms are placed posterior to the medial border of the tibia which is defined by palpation, and along the medial weight bearing axis of the foot from the load area of the heel to the load area of the first metatarsal head. A similar examination was performed with extended knee.

Supplemental figure 2. Photograph illustrating goniometric placement on the lateral side while performing the two-hand examination technique of ankle dorsiflexion with extended knee. The examiner performing the ankle dorsiflexion locks the hindfoot in varus, avoids midfoot abduction and applies dorsiflexion force to the whole foot, whilst still exposing the lateral aspect of the foot for consistent goniometer placement. The goniometer arms are placed along the fibula which is defined by palpation, and along the lateral weight bearing axis of the foot from the load area of the heel to the load area of the fifth metatarsal head. A similar examination was performed with flexed knee.
