# Supplementary figures and images for: Does the Side Matter? Medial vs Lateral Ankle Dorsiflexion Measurements During the Silfverskiöld Test in Children
Source: Foot Ankle Int. 2025 Jul 20;46(10):1176–83. doi: 10.1177/10711007251351317 (PMC12534877; doi:10.1177/10711007251351317)

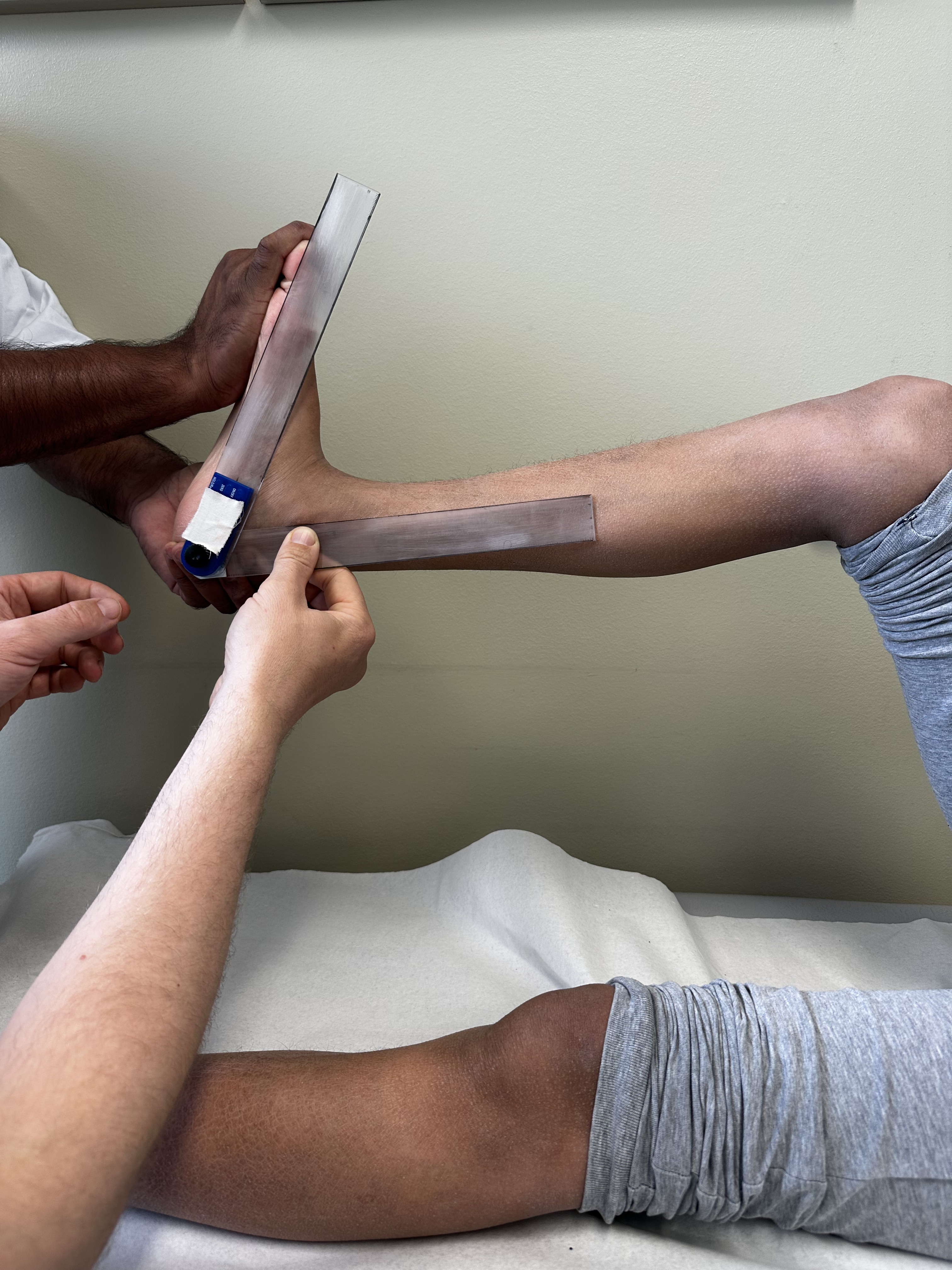

Supplement: sj-jpg-2-fai-10.1177_10711007251351317 – Supplemental material for Does the Side Matter? Medial vs Lateral Ankle Dorsiflexion Measurements During the Silfverskiöld Test in Children [file sj-jpg-2-fai-10.1177_10711007251351317.jpg]

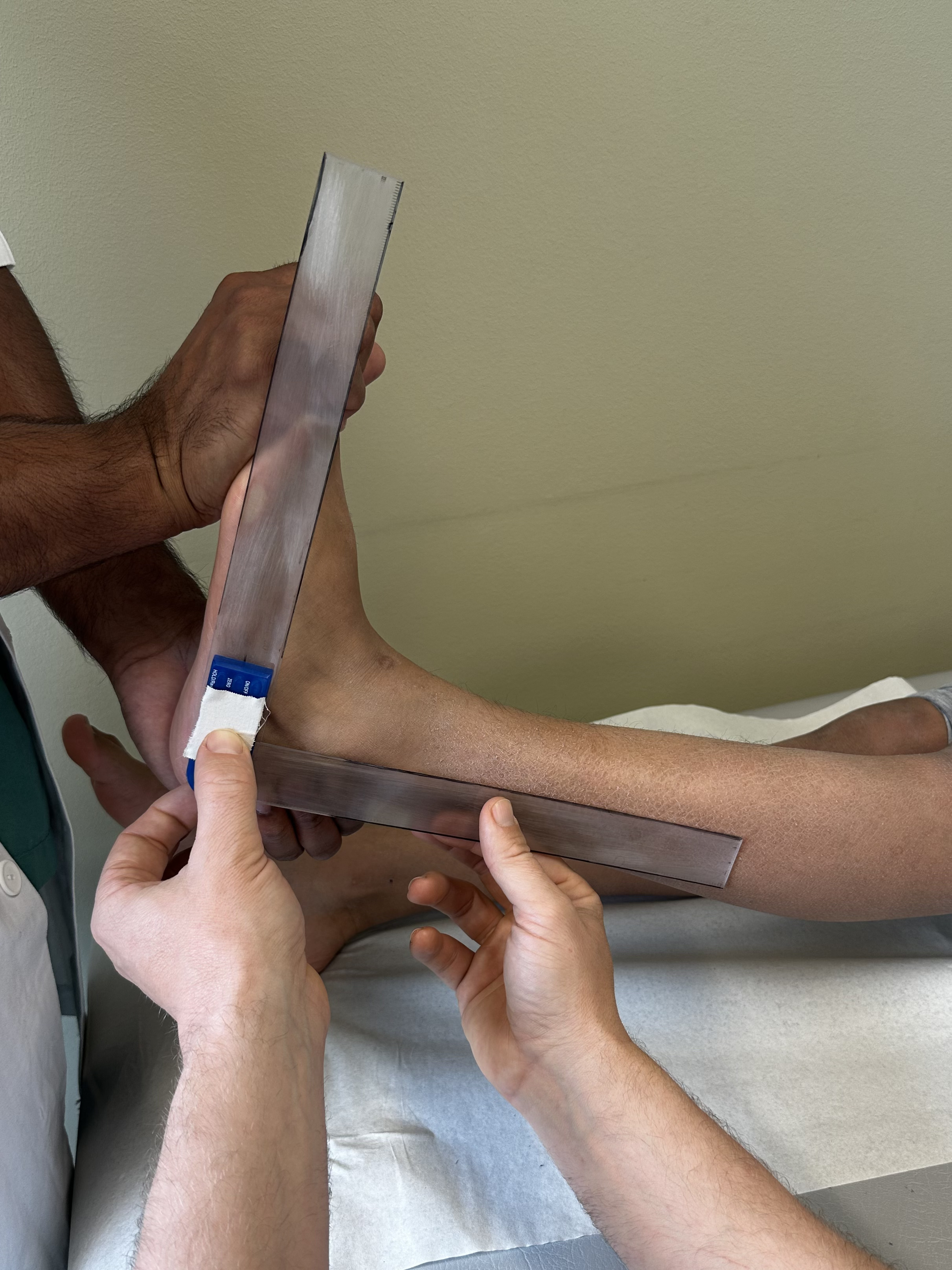

Supplement: sj-jpg-3-fai-10.1177_10711007251351317 – Supplemental material for Does the Side Matter? Medial vs Lateral Ankle Dorsiflexion Measurements During the Silfverskiöld Test in Children [file sj-jpg-3-fai-10.1177_10711007251351317.jpg]
